# Interactive Effects of HDL Cholesterol and hs-CRP in Relation to Cardiometabolic Risk Clustering Among Middle-Aged Adults

**DOI:** 10.3390/medicina62061096

**Published:** 2026-06-05

**Authors:** Heeyoung Hwang, Gitak Bae, Kyeongmin Jang

**Affiliations:** 1 Myongji St. Mary’s Hospital, Seoul 07417, Republic of Korea; kyo20107@gmail.com; 2 Seoul Metropolitan Government-Seoul National University Boramae Medical Center, Seoul 07061, Republic of Korea; kanu0520@gmail.com; 3 Department of Nursing, College of Health Sciences, Daejin University, Pocheon-si 11159, Republic of Korea

**Keywords:** cardiometabolic risk clustering, HDL cholesterol, hs-CRP, inflammation, lipid metabolism, middle-aged adults, KNHANES, cardiovascular risk

## Abstract

*Background and Objectives*: Cardiometabolic risk factors frequently cluster and substantially increase the risk of cardiovascular disease. While high-density lipoprotein cholesterol (HDL-C) and systemic inflammation, measured by high-sensitivity C-reactive protein (hs-CRP), are independently associated with cardiometabolic risk, whether systemic inflammation modifies the association between HDL-C and cardiometabolic risk clustering (CMRC) remains unclear. This study aimed to examine the independent associations of HDL-C and hs-CRP with CMRC and to evaluate the multiplicative product interaction between HDL-C and natural log-transformed hs-CRP in relation to CMRC among middle-aged adults. *Materials and Methods*: This cross-sectional study used data from 2283 adults aged 40–64 years who participated in the 2024 Korea National Health and Nutrition Examination Survey. CMRC was defined as the presence of ≥2 cardiometabolic risk factors. HDL-C and hs-CRP were analyzed as continuous variables. Complex sample logistic regression analyses were performed to estimate adjusted odds ratios (ORs) and 95% confidence intervals (CIs), including a multiplicative product interaction term calculated as HDL-C × ln hs-CRP. *Results*: The prevalence of CMRC was 46.4%. HDL-C was inversely associated with CMRC (OR = 0.946, 95% CI = 0.937–0.956, *p* < 0.001), whereas ln hs-CRP was positively associated with CMRC (OR = 1.224, 95% CI = 1.020–1.468, *p* = 0.030). The HDL-C × ln hs-CRP product interaction term was significantly associated with CMRC (OR = 1.005, 95% CI = 1.001–1.009, *p* = 0.008). This finding indicates that the association between HDL-C and CMRC may vary across levels of ln hs-CRP, but it does not indicate a direct association between HDL-C and ln hs-CRP. *Conclusions*: HDL-C and ln hs-CRP were independently associated with CMRC. The significant HDL-C × ln hs-CRP product interaction term suggests possible statistical effect modification of the HDL-C–CMRC association by systemic inflammatory status. These findings should be interpreted cautiously and do not establish a causal or mechanistic relationship.

## 1. Introduction

Cardiometabolic abnormalities, including abdominal obesity, hypertension, dyslipidemia, and impaired glucose metabolism, are major contributors to cardiovascular disease and premature mortality worldwide [1]. These risk factors often cluster rather than occur in isolation, and such aggregation has important implications for cardiometabolic risk stratification and the identification of biologically meaningful subphenotypes [2]. Recent evidence further suggests that cardiometabolic clustering reflects shared pathophysiological processes, particularly insulin resistance, chronic low-grade inflammation, and disturbed lipid metabolism, which interact to promote progression toward overt disease [3]. From a clinical and public health perspective, focusing on clustered risk profiles rather than single abnormalities may improve prevention and early identification of individuals at elevated risk [2]. This issue is especially relevant in middle-aged adults, in whom cardiometabolic risk commonly accumulates before the onset of clinically manifest cardiovascular disease, making this life stage an important window for risk assessment and intervention [4].

High-density lipoprotein cholesterol (HDL-C) has traditionally been viewed as a protective factor in cardiometabolic health because HDL participates in reverse cholesterol transport and also exerts anti-inflammatory and antioxidant effects [5]. However, increasing evidence indicates that the clinical relevance of HDL cannot be explained by HDL-C concentration alone. Instead, HDL functionality, particularly cholesterol efflux capacity, may be more closely related to cardiovascular risk than circulating HDL-C levels per se [6]. In addition, inflammatory and metabolic disturbances can alter HDL composition and impair its protective properties, giving rise to the concept of dysfunctional HDL [7]. Under these conditions, HDL may lose its expected atheroprotective effects and become less effective in modulating oxidative stress and inflammation [7].

In parallel, high-sensitivity C-reactive protein (hs-CRP) is widely used as a marker of systemic low-grade inflammation and has been consistently associated with cardiovascular risk [8]. Chronic inflammation is now recognized as a central component of atherosclerotic cardiovascular disease and is closely linked to endothelial dysfunction, metabolic dysregulation, and progression of cardiometabolic abnormalities [8,9]. Because inflammation can influence lipoprotein composition and function, it is biologically plausible that the association between HDL-C and cardiometabolic risk may vary according to inflammatory status. Nevertheless, most previous studies have evaluated HDL-C and hs-CRP separately, rather than examining their potential combined or interactive relationship. As a result, evidence remains limited regarding whether systemic inflammation modifies the association between HDL-C and cardiometabolic risk clustering in the general population.

This issue may be especially important in middle-aged adults. Cardiometabolic abnormalities often accumulate during midlife, and risk profiles established during this period may shape subsequent cardiovascular outcomes. Therefore, identifying combined biomarker patterns associated with clustered cardiometabolic risk in middle-aged adults may improve early risk stratification and support more timely preventive strategies. In this regard, nationally representative data are particularly valuable because they allow these associations to be examined beyond selected clinical groups. The Korea National Health and Nutrition Examination Survey (KNHANES), which uses a complex, multistage probability sampling design and standardized health assessments, provides an appropriate framework for evaluating these relationships at the population level [10,11].

Therefore, the present study aimed to investigate the independent associations of HDL-C and hs-CRP with cardiometabolic risk clustering in middle-aged Korean adults and to evaluate whether systemic inflammation statistically modified the association between HDL-C and CMRC. Specifically, we examined the association of HDL-C with CMRC, the association of ln hs-CRP with CMRC, and the multiplicative product interaction term between HDL-C and ln hs-CRP in relation to CMRC.

## 2. Materials and Methods

### 2.1. Study Design and Participants

This study was a cross-sectional secondary analysis using data from the 2024 Korea National Health and Nutrition Examination Survey (KNHANES), a nationally representative survey conducted by the Korea Disease Control and Prevention Agency [12]. KNHANES is a government-designated national surveillance system based on the National Health Promotion Act and uses a complex, multistage, stratified cluster sampling design to obtain representative data on the health and nutritional status of the non-institutionalized Korean population. In the ninth cycle (2022–2024), the survey was conducted year-round from January to December, with 192 survey districts and 4800 households sampled annually. All household members aged 1 year or older in selected households were eligible for participation [12].

The present study used the 2024 survey data, which were released in December 2025. Initially, 6997 participants were identified from the 2024 KNHANES dataset. Of these, 2317 individuals aged under 40 years and 1951 individuals aged 65 years or older were excluded to restrict the analysis to middle-aged adults. In addition, 446 participants with missing values in key variables required for the analysis, including blood test variables and cardiometabolic risk-related indicators, were excluded. Therefore, a total of 2283 middle-aged adults aged 40–64 years were included in the final analysis.

### 2.2. Outcome Variable: Cardiometabolic Risk Clustering

The primary outcome of this study was cardiometabolic risk clustering (CMRC). CMRC was defined as the presence of two or more cardiometabolic risk factors, based on commonly used clinical and epidemiological criteria [13]. In this study, cardiometabolic risk factors were defined as abdominal obesity, hypertension, hyperglycemia or diabetes, and hypertriglyceridemia, based on commonly used clinical criteria [14]. Low HDL cholesterol was not included in the clustering definition to avoid conceptual overlap with the primary exposure variable. Therefore, CMRC in this study should be interpreted as a modified cardiometabolic risk clustering index designed to avoid overlap with HDL-C as the primary exposure, rather than as a direct equivalent of the conventional metabolic syndrome definition. Abdominal obesity was defined as a waist circumference of at least 90 cm in men and at least 85 cm in women. Hypertension was defined as a systolic blood pressure of at least 130 mmHg, a diastolic blood pressure of at least 85 mmHg, or current use of antihypertensive medication. Hyperglycemia or diabetes was defined as a fasting plasma glucose level of at least 100 mg/dL, an HbA1c level of at least 6.5%, or physician-diagnosed diabetes. Hypertriglyceridemia was defined as a triglyceride level of at least 150 mg/dL. Low HDL cholesterol was defined as an HDL cholesterol level below 40 mg/dL in men and below 50 mg/dL in women. Each participant was classified according to the number of cardiometabolic risk factors present, and was subsequently categorized into one of two groups: those with fewer than two risk factors and those with two or more risk factors. This binary variable was used as the dependent variable in all analyses.

### 2.3. Exposure Variables: HDL Cholesterol and hs-CRP

The primary exposure variables in this study were serum high-density lipoprotein cholesterol (HDL-C) and high-sensitivity C-reactive protein (hs-CRP). These biomarkers were obtained from blood samples collected during the health examination component of the Korea National Health and Nutrition Examination Survey, which follows standardized protocols for specimen collection and laboratory analysis. Fasting blood samples were collected from participants and analyzed using certified laboratory procedures.

HDL-C levels were treated as a continuous variable and are expressed in mg/dL. hs-CRP levels were also analyzed as a continuous variable and are expressed in mg/L. Because hs-CRP demonstrated a right-skewed distribution, natural logarithmic transformation using base e was applied prior to regression analyses. For descriptive analyses, hs-CRP values are presented in the original scale as mg/L.

To examine whether systemic inflammation statistically modified the association between HDL-C and CMRC, a multiplicative product interaction term was created by multiplying HDL-C by natural log-transformed hs-CRP. This term is expressed as HDL-C × ln hs-CRP and entered into the multivariable complex sample logistic regression model together with the main effects of HDL-C and ln hs-CRP. The OR for this product term represents the association between the HDL-C × ln hs-CRP term and CMRC, not a direct association between HDL-C and ln hs-CRP. Therefore, the interaction term was interpreted as evidence of possible statistical effect modification and was not considered sufficient, by itself, to establish a biological mechanism [15,16].

### 2.4. Covariates: Sociodemographic and Health-Related Variables

Covariates were selected based on previous literature and their potential confounding effects on the association between biomarkers and cardiometabolic risk clustering. Sociodemographic variables included age (years), sex (male or female), residential area (urban or rural), educational level (≤primary school, middle school, high school, or ≥college), and household income (low, mid-low, mid-high, or high).

Health-related behavioral variables included alcohol use, current smoking status, aerobic physical activity, and strength exercise. Alcohol use was defined as any alcohol consumption within the past month and was categorized as yes or no. Current smoking status was categorized as yes or no. Aerobic physical activity was classified according to whether participants met the recommended level of at least 150 min of moderate-intensity physical activity per week, and was categorized as yes or no. Strength exercise was categorized according to whether participants met the WHO recommendation for muscle-strengthening activity. Participants who reported muscle-strengthening exercise on two or more days per week were classified as meeting the recommendation, whereas those who reported no muscle-strengthening exercise or exercise on only one day per week were classified as not meeting the recommendation [17].

All covariates were obtained from the health interview and health behavior survey components of the Korea National Health and Nutrition Examination Survey, which were conducted using standardized procedures.

### 2.5. Statistical Analysis

All analyses were performed using IBM SPSS Statistics version 30.0 for Windows (IBM Corp., Armonk, NY, USA). Because the Korea National Health and Nutrition Examination Survey uses a complex, multistage, stratified cluster sampling design, all analyses incorporated sampling weights, stratification variables, and primary sampling units to obtain nationally representative estimates. Categorical variables are presented as unweighted frequencies and weighted percentages, and continuous variables are expressed as weighted means with standard errors (SEs). In complex sample analyses, SEs indicate the precision of weighted population estimates rather than the dispersion of individual-level observations. Group differences according to CMRC status were examined using the Rao–Scott chi-square test for categorical variables and complex sample general linear models for continuous variables.

Complex sample logistic regression analyses were performed to examine the independent associations of HDL-C and hs-CRP with CMRC and to assess the HDL-C × ln hs-CRP product interaction term. HDL-C and natural log-transformed hs-CRP were entered as continuous variables. A multiplicative product interaction term, calculated as HDL-C × ln hs-CRP, was included in the model to assess whether the association between HDL-C and CMRC varied according to ln hs-CRP levels. The OR for this product term was not interpreted as indicating a direct association between HDL-C and ln hs-CRP. The multivariable model was adjusted for age, sex, residential area, educational level, household income, alcohol use, current smoking status, aerobic physical activity, and strength exercise. To improve the interpretability of the product interaction term, additional stratified analyses were conducted according to hs-CRP tertiles. Sex-stratified and obesity-stratified analyses were also performed to examine whether these associations differed by sex and obesity status. Obesity was defined as BMI ≥ 25 kg/m^2^. As a sensitivity analysis, the main logistic regression model was repeated after excluding participants with hs-CRP levels >10 mg/L. Adjusted odds ratios (ORs), 95% confidence intervals (CIs), and two-sided *p*-values are reported, with statistical significance set at *p* < 0.05.

### 2.6. Ethical Considerations

The Korea National Health and Nutrition Examination Survey was conducted in accordance with the Declaration of Helsinki and approved by the Institutional Review Board of the Korea Disease Control and Prevention Agency (approval number: 2022-11-16-R-03). All participants provided informed consent prior to participation. The present study used publicly available de-identified data and was therefore exempt from additional institutional review board approval.

## 3. Results

### 3.1. General Characteristics of the Study Participants

A total of 2283 participants were included in the analysis. The weighted mean age was 52.30 years (SE = 0.23), and 53.5% were male. Most participants resided in urban areas (83.4%), and more than half had a college-level education or higher (53.1%). Regarding health behaviors, 61.5% reported alcohol use, 21.3% were current smokers, and 43.3% engaged in aerobic physical activity. The weighted mean HDL-C level was 56.96 mg/dL (SE = 0.36), and the weighted mean hs-CRP level was 1.28 mg/L (SE = 0.06). Overall, 46.4% of participants had cardiometabolic risk clustering (CMRC ≥2 risk factors) (Table 1).

### 3.2. Differences According to Cardiometabolic Risk Clustering

Participants with CMRC ≥2 risk factors were significantly older than those with CMRC <2 (54.1 vs. 50.9 years, *p* = 0.001) and had a higher proportion of males (66.5% vs. 42.3%, *p* < 0.001). Educational level differed significantly between groups, with a higher proportion of lower education levels in the CMRC ≥2 group (*p* < 0.001). In terms of health behaviors, alcohol use (65.1% vs. 58.4%, *p* = 0.004), current smoking (25.9% vs. 17.3%, *p* < 0.001), and not meeting the strength exercise recommendation (81.0% vs. 74.3%, *p* < 0.001) were more prevalent among participants with CMRC ≥2. For continuous variables, the CMRC ≥2 group had lower HDL-C levels (50.94 vs. 62.16 mg/dL, *p* < 0.001) and higher hs-CRP levels (1.55 vs. 1.04 mg/L, *p* < 0.001) than the CMRC <2 group. No significant differences were observed for residence, household income, or aerobic physical activity (Table 2).

### 3.3. Factors Associated with Cardiometabolic Risk Clustering

In the complex sample logistic regression analysis, HDL-C was inversely associated with cardiometabolic risk clustering (OR = 0.946, 95% CI = 0.937–0.956, *p* < 0.001), whereas natural log-transformed hs-CRP was positively associated with CMRC (OR = 1.224, 95% CI = 1.020–1.468, *p* = 0.030). The HDL-C × ln hs-CRP product interaction term was significantly associated with CMRC (OR = 1.005, 95% CI = 1.001–1.009, *p* = 0.008). This result indicates that the association between HDL-C and CMRC differed modestly according to ln hs-CRP levels. Because the coefficient for the product term was positive, the inverse association between HDL-C and CMRC appeared to become slightly weaker as ln hs-CRP increased. This finding should not be interpreted as evidence of a direct association between HDL-C and ln hs-CRP. Age (OR = 1.017, 95% CI = 1.002–1.033, *p* = 0.026) and male sex (OR = 1.850, 95% CI = 1.443–2.370, *p* < 0.001) were also significantly associated with increased odds of CMRC. Lower educational attainment (≤primary vs. ≥college) was associated with higher odds of CMRC. Alcohol use was associated with lower odds of CMRC (OR = 0.731, *p* = 0.007), whereas not meeting the strength exercise recommendation was associated with higher odds of CMRC (OR = 1.701, *p* < 0.001). Other variables, including residence, household income, current smoking, and aerobic physical activity, were not significantly associated with CMRC (Table 3).

To improve the interpretability of the HDL-C × ln hs-CRP product interaction term, additional analyses were conducted according to hs-CRP tertiles. The inverse association between HDL-C and CMRC was observed across all hs-CRP tertiles; however, the magnitude of the association was strongest in the lowest hs-CRP tertile and weaker in the middle and highest tertiles. The adjusted ORs for HDL-C were 0.942 (95% CI = 0.927–0.958, *p* < 0.001) in the lowest tertile, 0.962 (95% CI = 0.945–0.978, *p* < 0.001) in the middle tertile, and 0.963 (95% CI = 0.949–0.977, *p* < 0.001) in the highest tertile (Table 4). These findings suggest modest variation in the HDL-C–CMRC association across inflammatory strata and support cautious interpretation of the product interaction term as statistical effect modification.

In the sensitivity analysis excluding 22 participants with hs-CRP levels >10 mg/L, the results were generally consistent with the main analysis. HDL-C remained inversely associated with CMRC (OR = 0.943, 95% CI = 0.932–0.954, *p* < 0.001), ln hs-CRP remained positively associated with CMRC (OR = 1.221, 95% CI = 1.021–1.451, *p* = 0.036), and the HDL-C × ln hs-CRP product interaction term remained statistically significant (OR = 1.010, 95% CI = 1.004–1.016, *p* = 0.002) (Supplementary Table S1).

In sex-stratified analyses, HDL-C remained inversely associated with CMRC in both men and women, and the HDL-C × ln hs-CRP product interaction term appeared more evident among women (Supplementary Table S2). Obesity-stratified analyses showed that HDL-C remained inversely associated with CMRC in both participants with obesity and those without obesity, while the HDL-C × ln hs-CRP product interaction term appeared more evident among participants without obesity (Supplementary Table S3).

## 4. Discussion

The present study examined the independent associations of HDL-C and hs-CRP with cardiometabolic risk clustering (CMRC) and evaluated whether ln hs-CRP statistically modified the association between HDL-C and CMRC in a nationally representative population. The main findings were that HDL-C was inversely associated with CMRC, whereas ln hs-CRP was positively associated with CMRC. In addition, the HDL-C × ln hs-CRP multiplicative product interaction term was significantly associated with CMRC. This finding suggests that the inverse association between HDL-C and CMRC may vary according to systemic inflammatory status. Importantly, this result should not be interpreted as evidence of a direct association between HDL-C and ln hs-CRP. However, given the cross-sectional design and the small magnitude of the interaction term, this finding should be interpreted cautiously as possible effect modification rather than evidence of a causal or mechanistic relationship.

The inverse association between HDL-C and CMRC observed in this study is consistent with previous evidence demonstrating the protective role of HDL in cardiovascular health. HDL has long been recognized for its role in reverse cholesterol transport and its anti-inflammatory and antioxidant properties [18]. However, recent studies have emphasized that HDL functionality, rather than HDL-C concentration alone, plays a critical role in determining cardiovascular risk [19,20]. Cholesterol efflux capacity, a key functional property of HDL, has been shown to be more strongly associated with cardiovascular outcomes than HDL-C levels per se [20]. In addition, HDL particles are heterogeneous and may exhibit functional impairment under adverse metabolic conditions, further supporting the importance of HDL quality over quantity [19].

In contrast, hs-CRP was positively associated with CMRC, which is in line with extensive evidence linking chronic low-grade inflammation to cardiometabolic disease. Systemic inflammation plays a central role in the development of insulin resistance, endothelial dysfunction, and atherosclerosis, thereby contributing to the clustering of cardiometabolic risk factors [18]. hs-CRP has been widely used as a biomarker of inflammation and is consistently associated with increased cardiovascular risk and adverse outcomes. These findings reinforce the importance of inflammatory pathways in cardiometabolic disease and highlight the need to consider inflammation alongside traditional metabolic risk factors.

A notable finding of this study was that the HDL-C × ln hs-CRP multiplicative product interaction term was statistically significant in relation to CMRC. This result suggests possible statistical effect modification, meaning that the association between HDL-C and CMRC may differ according to ln hs-CRP levels. The positive coefficient of the product term indicates that the inverse association between HDL-C and CMRC was slightly attenuated at higher ln hs-CRP levels. However, the OR for the product term should not be interpreted as indicating a direct association between HDL-C and ln hs-CRP. Moreover, because the magnitude of the interaction was modest and the study design was cross-sectional, this finding should be interpreted cautiously and should not be considered evidence of a definitive biological mechanism. To improve the interpretability of the product interaction term, we conducted additional analyses stratified by hs-CRP tertiles. HDL-C remained inversely associated with CMRC across all tertiles, but the magnitude of the association was strongest in the lowest hs-CRP tertile and modestly weaker in the middle and highest tertiles. This pattern supports possible effect modification by systemic inflammatory status; however, the differences across tertiles were not large, and the findings should not be interpreted as evidence of a large individual-level clinical effect. The observed pattern is biologically plausible in light of the concept of inflammation-related HDL dysfunction, although HDL functionality was not directly measured in the present study. Under inflammatory conditions, HDL particles may undergo structural and compositional changes that could impair antioxidant and anti-inflammatory functions [7,21]. However, the present study cannot determine whether such functional changes occurred. Therefore, interpretations related to dysfunctional HDL remain speculative. Future studies incorporating direct measures of HDL function, such as cholesterol efflux capacity or anti-inflammatory activity, are needed to clarify whether inflammation-related HDL dysfunction explains this association. Exploratory sex- and obesity-stratified analyses suggested that the HDL-C × ln hs-CRP product interaction term may be more evident among women and among participants without obesity; however, these findings should be interpreted cautiously because the analyses were exploratory and not designed to establish subgroup-specific mechanisms.

Recent studies have further supported the relevance of considering lipid metabolism and systemic inflammation together in cardiometabolic risk assessment. Evidence suggests that combined consideration of lipid and inflammatory components may provide additional context for cardiometabolic risk assessment compared with single biomarkers alone. For example, studies examining HDL functionality and inflammatory status have demonstrated that the predictive value of HDL-related measures is modified by systemic inflammation [22]. Additionally, epidemiological studies have reported that HDL-C levels alone may not consistently predict cardiovascular outcomes, particularly at extreme levels, further emphasizing the importance of functional and contextual interpretation [23,24]. These findings align with the present results and suggest that the observed interaction may reflect possible interplay between lipid metabolism and inflammation, although the underlying mechanisms could not be directly examined in this study.

From a clinical perspective, the present findings have important implications. Traditional risk assessment strategies often consider lipid profiles and inflammatory markers separately. However, the results of this study suggest that the association between HDL-C and CMRC may be interpreted more cautiously when systemic inflammatory burden is elevated. Instead, an integrated approach that considers both lipid and inflammatory biomarkers may provide a more comprehensive evaluation of cardiometabolic risk. Such an approach may help refine cardiometabolic risk assessment, especially in middle-aged populations, although its predictive utility should be confirmed in future longitudinal studies.

The findings of this study should also be interpreted in the broader context of cardiometabolic disease prevention. Middle-aged adults represent a critical period for the development and accumulation of cardiometabolic risk factors. During this stage, subclinical metabolic abnormalities may progress toward overt disease if not appropriately managed. Therefore, identifying individuals with both adverse lipid profiles and elevated inflammatory markers may provide an opportunity for early intervention. Lifestyle modifications, including dietary changes and physical activity, have been shown to improve HDL functionality and reduce inflammation, suggesting potential synergistic benefits [25,26].

Alcohol use was inversely associated with CMRC in the adjusted model. This finding should be interpreted cautiously. Alcohol use was assessed as a binary variable based on any alcohol consumption within the past month, which did not capture drinking frequency, amount, binge drinking, beverage type, or former drinking status. Therefore, the observed inverse association may reflect residual confounding, exposure misclassification, or heterogeneity in drinking patterns rather than a protective effect of alcohol consumption.

Several limitations should be considered. First, the cross-sectional design precludes causal inference, and longitudinal studies are needed to confirm temporality. Second, residual confounding cannot be entirely excluded because information on diet, detailed alcohol consumption patterns, medication use, and comorbid conditions was limited or not fully incorporated into the analysis. Third, HDL functionality was not directly measured; therefore, interpretations related to dysfunctional HDL remain indirect and should be regarded as speculative. Fourth, low HDL-C was excluded from the CMRC definition to avoid conceptual overlap with HDL-C as the primary exposure variable. Although this approach was methodologically necessary for the present research question, the resulting CMRC definition differs from the conventional metabolic syndrome definition and may have underestimated the overall severity of cardiometabolic clustering. We did not perform a sensitivity analysis using the full metabolic syndrome definition because doing so would have reintroduced HDL-C into the outcome and created overlap between the exposure and dependent variable, thereby reducing the interpretability of the HDL-C–CMRC association. Fifth, hs-CRP was measured at a single time point, and transient inflammation may have influenced the results. To address this concern, a sensitivity analysis excluding participants with hs-CRP levels >10 mg/L was performed, and the main findings were generally consistent. Finally, the findings may not be generalizable beyond middle-aged Korean adults. In addition, the interaction analysis was based on a multiplicative product term in a regression model; therefore, the OR for the HDL-C × ln hs-CRP term should be interpreted only as evidence of statistical effect modification and not as evidence of a direct association between HDL-C and ln hs-CRP.

Despite these limitations, this study has several strengths, including the use of nationally representative data and the assessment of possible statistical effect modification of the HDL-C–CMRC association by ln hs-CRP. These findings provide important insights into the interplay between lipid metabolism and systemic inflammation in cardiometabolic risk.

## 5. Conclusions

HDL-C was inversely associated with cardiometabolic risk clustering, whereas ln hs-CRP showed a positive association. The HDL-C × ln hs-CRP multiplicative product interaction term was statistically significant in relation to CMRC, suggesting possible statistical effect modification of the HDL-C–CMRC association by systemic inflammatory status. This finding does not indicate a direct association between HDL-C and ln hs-CRP and should not be interpreted as evidence of a causal or mechanistic relationship. Future longitudinal studies are needed to determine whether combined lipid and inflammatory biomarker assessment improves clinical risk prediction.

## Figures and Tables

**Table 1:** General characteristics of the study participants (*n* = 2283).

Variable	Category	n	Weighted % or Mean ± SE
Age (years)		2283	52.30 ± 0.23
Sex	Male	1038	53.5
	Female	1245	46.5
Residence	Urban	1870	83.4
	Rural	413	16.6
Education	≤Primary school	152	3.8
	Middle school	192	7.5
	High school	830	35.6
	≥College	1109	53.1
Household income	Low	179	6.4
	Mid-low	464	20.1
	Mid-high	714	32.1
	High	926	41.4
Alcohol use	No	963	38.5
	Yes	1320	61.5
Current smoking	No	1863	78.7
	Yes	420	21.3
Aerobic PA	No	1376	56.7
	Yes	907	43.3
Strength exercise	<2 days/week	1777	77.4
	≥2 days/week	506	22.6
HDL-C (mg/dL)		2283	56.96 ± 0.36
hs-CRP (mg/L)		2283	1.28 ± 0.06
CMRC	<2 risk factors	1260	53.6
	≥2 risk factors	1023	46.4

Note. Values are presented as unweighted frequency (n) and weighted percentage for categorical variables, and weighted mean ± standard error (SE) for continuous variables. In this complex sample analysis, SEs indicate the precision of weighted population estimates rather than the dispersion of individual observations. Percentages were weighted to account for the complex sampling design and may differ from unweighted proportions. HDL-C = high-density lipoprotein cholesterol; hs-CRP = high-sensitivity C-reactive protein; PA = physical activity; CMRC = cardiometabolic risk clustering. CMRC was defined as the presence of ≥2 cardiometabolic risk factors (abdominal obesity, hypertension, hyperglycemia or diabetes, and hypertriglyceridemia). All estimates were obtained using complex sample procedures accounting for stratification, clustering, and sampling weights.

**Table 2:** Differences in participant characteristics according to cardiometabolic risk clustering.

Variable	Category	CMRC <2 (Mean ± SE or %)	CMRC ≥2 (Mean ± SE or %)	F (Rao–Scott χ^2^)	*p*
Age (years)		50.9 ± 0.30	54.1 ± 0.31	10.12	0.001
Sex	Male	42.3	66.5	118.13	<0.001
	Female	57.7	33.5		
Residence	Urban	84.7	81.9	2.16	0.144
	Rural	15.3	18.1		
Education	≤Primary school	2.3	5.5	7.01	<0.001
	Middle school	6.3	8.8		
	High school	34.8	36.6		
	≥College	56.5	49.1		
Household income	Low	6.6	6.3	0.18	0.900
	Mid-low	19.5	20.9		
	Mid-high	32.3	31.8		
	High	41.6	41.1		
Alcohol use	No	41.6	34.9	8.47	0.004
	Yes	58.4	65.1		
Current smoking	No	82.7	74.1	19.51	<0.001
	Yes	17.3	25.9		
Aerobic PA	No	54.8	58.9	2.68	0.103
	Yes	45.2	41.1		
Strength exercise	<2 days/week	74.3	81.0	14.02	<0.001
	≥2 days/week	25.7	19.0		
HDL-C (mg/dL)		62.16 ± 0.47	50.94 ± 0.45	311.45	<0.001
hs-CRP (mg/L)		1.04 ± 0.07	1.55 ± 0.10	22.69	<0.001

Note. Values are presented as weighted percentages for categorical variables and weighted mean ± standard error (SE) for continuous variables. SEs indicate the precision of weighted population estimates from complex sample analyses and should not be interpreted as measures of individual-level dispersion. For categorical variables, F statistics were derived from the Rao–Scott adjusted chi-square test. For continuous variables, F statistics were obtained from complex sample general linear models. HDL-C = high-density lipoprotein cholesterol; hs-CRP = high-sensitivity C-reactive protein; PA = physical activity; CMRC = cardiometabolic risk clustering. CMRC was defined as the presence of ≥2 cardiometabolic risk factors (abdominal obesity, hypertension, hyperglycemia or diabetes, and hypertriglyceridemia). All analyses accounted for stratification, clustering, and sampling weights. Due to rounding, percentages may not sum exactly to 100.0%.

**Table 3:** Complex sample logistic regression for cardiometabolic risk clustering.

Variable	B	SE	OR	95% CI	*p*
HDL-C	−0.055	0.005	0.946	0.937–0.956	<0.001
ln hs-CRP	0.202	0.092	1.224	1.020–1.468	0.030
HDL-C × ln hs-CRP	0.005	0.002	1.005	1.001–1.009	0.008
Age	0.017	0.008	1.017	1.002–1.033	0.026
Male (vs. female)	0.615	0.126	1.850	1.443–2.370	<0.001
Residence (urban vs. rural)	0.040	0.179	1.041	0.730–1.481	0.825
Household income (low vs. high)	−0.217	0.219	0.804	0.522–1.240	0.324
Household income (mid-low vs. high)	−0.124	0.177	0.884	0.623–1.251	0.484
Household income (mid-high vs. high)	−0.067	0.132	0.936	0.720–1.212	0.610
Education (≤primary vs. college+)	1.129	0.286	3.092	1.758–5.435	<0.001
Education (middle vs. college+)	0.434	0.240	1.544	0.962–2.475	0.072
Education (high vs. college+)	0.232	0.118	1.261	1.000–1.593	0.050
Alcohol use (yes vs. no)	−0.314	0.115	0.731	0.582–0.917	0.007
Current smoking (yes vs. no)	0.098	0.147	1.103	0.825–1.476	0.505
Aerobic PA (yes vs. no)	−0.075	0.116	0.928	0.737–1.167	0.517
Strength exercise (<2 days/week vs. ≥2 days/week)	0.531	0.120	1.701	1.344–2.150	<0.001

Note. The outcome variable was cardiometabolic risk clustering (CMRC ≥2 risk factors). B = regression coefficient; SE = standard error; OR = odds ratio; CI = confidence interval; HDL-C = high-density lipoprotein cholesterol; hs-CRP = high-sensitivity C-reactive protein; PA = physical activity. Estimates were derived from complex sample logistic regression accounting for stratification, clustering, and sampling weights. Reference categories were female (sex), rural (residence), high (household income), college or higher (education), no alcohol use, no current smoking, meeting the aerobic physical activity recommendation, and meeting the strength exercise recommendation. Model fit: Nagelkerke R^2^ = 0.234; the overall model was significant (Wald F = 14.70, *p* < 0.001). hs-CRP was natural log-transformed using base e before inclusion in the regression model. The interaction term was a multiplicative product term calculated by multiplying HDL-C by ln hs-CRP (HDL-C × ln hs-CRP). The OR for this term represents the association between the product interaction term and CMRC, not a direct association between HDL-C and ln hs-CRP.

**Table 4:** Association between HDL-C and cardiometabolic risk clustering according to hs-CRP tertiles.

hs-CRP Tertile	hs-CRP Range, mg/L	n	HDL-C OR	95% CI	*p*
T1, lowest	0.2–0.4	902	0.942	0.927–0.958	<0.001
T2, middle	0.5–0.8	608	0.962	0.945–0.978	<0.001
T3, highest	0.9–61.6	773	0.963	0.949–0.977	<0.001

Note. The outcome variable was cardiometabolic risk clustering, defined as ≥2 risk factors. hs-CRP tertile groups were created based on the distribution of hs-CRP. Group sizes were not identical because of tied hs-CRP values. Each model estimated the association between HDL-C and CMRC within each hs-CRP tertile and was adjusted for age, sex, residence, household income, education, alcohol use, current smoking, aerobic physical activity, and strength exercise. HDL-C = high-density lipoprotein cholesterol; hs-CRP = high-sensitivity C-reactive protein; CMRC = cardiometabolic risk clustering; OR = odds ratio; CI = confidence interval.

## Data Availability

Publicly available datasets were analyzed in this study. These data can be found at https://knhanes.kdca.go.kr.

## Supplementary Materials

The following supporting information can be downloaded at https://www.mdpi.com/article/10.3390/medicina62061096/s1, Table S1. Sensitivity analysis excluding participants with hs-CRP levels >10 mg/L; Table S2. Sex-stratified analysis of the association between HDL-C, ln hs-CRP, and cardiometabolic risk clustering; Table S3. Obesity-stratified analysis of the association between HDL-C, ln hs-CRP, and cardiometabolic risk clustering.

## Abbreviations

The following abbreviations are used in this manuscript:

CI	Confidence interval
CMRC	Cardiometabolic risk clustering
HDL-C	High-density lipoprotein cholesterol
hs-CRP	High-sensitivity C-reactive protein
KDCA	Korea Disease Control and Prevention Agency
KNHANES	Korea National Health and Nutrition Examination Survey
OR	Odds ratio
SE	Standard error
