# Supplementary material for: Interactive Effects of HDL Cholesterol and hs-CRP in Relation to Cardiometabolic Risk Clustering Among Middle-Aged Adults
Source: Medicina (Kaunas). 2026 Jun 5;62(6):1096. doi: 10.3390/medicina62061096 (PMC13303969; doi:10.3390/medicina62061096)
Supplement: Supplementary file 1 [file medicina-62-01096-s001.zip › medicina-4300584-supplementary.pdf]

Supplementary Table S1. Sensitivity analysis excluding participants with hs-CRP levels >10 mg/L

Variable	B	SE	OR	95% CI	*p*
HDL-C	-0.059	0.006	0.943	0.932–0.954	<0.001
ln hs-CRP	0.199	0.091	1.221	1.021–1.451	0.036
HDL-C × ln hs-CRP	0.01	0.003	1.01	1.004–1.016	0.002
Age	0.02	0.008	1.02	1.005–1.036	0.009
Male (vs female)	0.59	0.124	1.805	1.410–2.304	<0.001
Residence (urban vs rural)	0.041	0.174	1.037	0.722–1.480	0.936
Household income (low vs high)	-0.225	0.213	0.799	0.524–1.217	0.292
Household income (mid-low vs high)	-0.138	0.172	0.871	0.620–1.222	0.424
Household income (mid-high vs high)	-0.055	0.139	0.946	0.719–1.244	0.693
Education (≤primary vs college+)	1.263	0.284	3.534	2.016–6.211	<0.001
Education (middle vs college+)	0.414	0.249	1.513	0.926–2.475	0.098
Education (high vs college+)	0.24	0.116	1.272	1.011–1.600	0.04
Alcohol use (yes vs no)	-0.308	0.109	0.691	0.577–0.921	0.01
Current smoking (yes vs no)	0.097	0.145	1.091	0.821–1.469	0.49
Aerobic PA (yes vs no)	-0.071	0.112	0.93	0.731–1.177	0.575
Strength exercise (<2 days/week vs ≥2 days/week)	0.486	0.122	1.626	1.277–2.070	<0.001

Note. The outcome variable was cardiometabolic risk clustering, defined as ≥2 risk factors. This sensitivity analysis excluded 22 participants with hs-CRP levels >10 mg/L. hs-CRP was natural log-transformed using base e before inclusion in the regression model. The interaction term was calculated as HDL-C × ln hs-CRP. OR = odds ratio; CI = confidence interval; HDL-C = high-density lipoprotein cholesterol; hs-CRP = high-sensitivity C-reactive protein; PA = physical activity.

Supplementary Table S2. Sex-stratified analysis of the association between HDL-C, ln hs-CRP, and cardiometabolic risk clustering

Variable	Men OR	95% CI	p	Women OR	95% CI	*p*
HDL-C	0.952	0.939–0.967	<0.001	0.938	0.925–0.951	<0.001
ln hs-CRP	1.114	0.867–1.432	0.395	1.435	1.034–1.990	0.031
HDL-C × ln hs-CRP	1.004	0.998–1.009	0.203	1.008	1.002–1.013	0.006

Note. The outcome variable was cardiometabolic risk clustering, defined as ≥2 risk factors. hs-CRP was natural log-transformed using base e before inclusion in the regression model. Each model was adjusted for age, residence, household income, education, alcohol use, current smoking, aerobic physical activity, and strength exercise. ORs were converted from the SPSS output because the output modeled CMRC <2 risk factors with CMRC ≥2 risk factors as the reference category. In models including an interaction term, the main effect of ln hs-CRP represents the conditional association when HDL-C equals zero and should therefore be interpreted cautiously. HDL-C = high-density lipoprotein cholesterol; hs-CRP = high-sensitivity C-reactive protein; CMRC = cardiometabolic risk clustering; OR = odds ratio; CI = confidence interval.

Supplementary Table S3. Obesity-stratified analysis of the association between HDL-C, ln hs-CRP, and cardiometabolic risk clustering

Variable	Obesity OR	95% CI	p	Non-obesity OR	95% CI	p
HDL-C	0.968	0.950–0.986	<0.001	0.95	0.936–0.964	<0.001
ln hs-CRP	1.243	0.946–1.632	0.117	1.237	0.938–1.631	0.131
HDL-C × ln hs-CRP	1.005	0.999–1.012	0.085	1.005	1.000–1.009	0.03

Note. Obesity was defined as BMI ≥25 kg/m^2. The outcome variable was cardiometabolic risk clustering, defined as ≥2 risk factors. hs-CRP was natural log-transformed using base e before inclusion in the regression model. Each model was adjusted for age, sex, residence, household income, education, alcohol use, current smoking, aerobic physical activity, and strength exercise. ORs were converted from the SPSS output because the output modeled CMRC <2 risk factors with CMRC ≥2 risk factors as the reference category. In models including an interaction term, the main effect of ln hs-CRP represents the conditional association when HDL-C equals zero and should therefore be interpreted cautiously. HDL-C = high-density lipoprotein cholesterol; hs-CRP = high-sensitivity C-reactive protein; CMRC = cardiometabolic risk clustering; OR = odds ratio; CI = confidence interval.
